# A Real-Time, Plate-Based BRET Assay for Detection of cGMP in Primary Cells

**DOI:** 10.3390/ijms23031908

**Published:** 2022-02-08

**Authors:** Adam L. Valkovic, Martina Kocan, Brad Hoare, Sarah Marshall, Daniel J. Scott, Ross A. D. Bathgate

**Affiliations:** 1 Florey Institute of Neuroscience and Mental Health, The University of Melbourne, Parkville, VIC 3052, Australia; adam.valkovic@florey.edu.au (A.L.V.); martina.kocan@unimelb.edu.au (M.K.); brad.hoare@florey.edu.au (B.H.); daniel.scott@florey.edu.au (D.J.S.); 2 The Ritchie Centre, Department of Obstetrics and Gynecology, Monash University, Clayton, VIC 3168, Australia; sarah.marshall@monash.edu; 3 Department of Biochemistry and Pharmacology, The University of Melbourne, Parkville, VIC 3052, Australia

**Keywords:** cGMP, BRET, biosensor, cell signalling

## Abstract

Cyclic guanosine monophosphate (cGMP) is a second messenger involved in the regulation of numerous physiological processes. The modulation of cGMP is important in many diseases, but reliably assaying cGMP in live cells in a plate-based format with temporal resolution is challenging. The Förster/fluorescence resonance energy transfer (FRET)-based biosensor cGES-DE5 has a high temporal resolution and high selectivity for cGMP over cAMP, so we converted it to use bioluminescence resonance energy transfer (BRET), which is more compatible with plate-based assays. This BRET variant, called CYGYEL (cyclic GMP sensor using YFP-PDE5-Rluc8), was cloned into a lentiviral vector for use across different mammalian cell types. CYGYEL was characterised in HEK293T cells using the nitric oxide donor diethylamine NONOate (DEA), where it was shown to be dynamic, reversible, and able to detect cGMP with or without the use of phosphodiesterase inhibitors. In human primary vascular endothelial and smooth muscle cells, CYGYEL successfully detected cGMP mediated through either soluble or particulate guanylate cyclase using DEA or C-type natriuretic peptide, respectively. Notably, CYGYEL detected differences in kinetics and strength of signal both between ligands and between cell types. CYGYEL remained selective for cGMP over cAMP, but this selectivity was reduced compared to cGES-DE5. CYGYEL streamlines the process of cGMP detection in plate-based assays and can be used to detect cGMP activity across a range of cell types.

## 1. Introduction

Cyclic guanosine monophosphate (cGMP) is a second messenger involved in the regulation of many physiological processes including cardiovascular homeostasis, smooth muscle tone, blood pressure, platelet aggregation, memory, learning, and sensory transduction [1,2,3]. cGMP is synthesised from guanosine triphosphate (GTP) by two forms of guanylate cyclase (GC): soluble GCs (sGCs), which are cytosolic enzymes activated by nitric oxide (NO) and carbon monoxide [4]; and particulate GCs (pGCs), which are membrane receptors activated by natriuretic peptides and some intestinal peptides [5]. cGMP signals via protein kinase G (PKG) and cyclic nucleotide-gated ion channels, and also modulates the activity of some phosphodiesterases (PDEs) [6]. cGMP is degraded by PDE-mediated hydrolysis [6].

Modulation of cGMP activity has therapeutic value for several disorders. For example, NO donors such as glyceryl trinitrate are used to treat angina [7], the sGC stimulator riociguat is used for treatment of pulmonary arterial hypertension and chronic thromboembolic pulmonary hypertension [8], PDE5 inhibitors such as sildenafil and vardenafil are used to treat erectile dysfunction [9], and agonists for GC-C (a type of pGC) such as linaclotide and plecanatide are used to treat irritable bowel syndrome with constipation [10]. Natriuretic peptides are also under investigation for the treatment of cardiovascular disorders such as heart failure [11], and drug discovery targeting cGMP signalling cascades is under continued investigation for the treatment of a variety of other disorders and diseases [12,13,14,15,16].

cGMP is usually measured in populations of cells in a plate-based format using traditional end point signalling assays that involve the detection of cGMP in cell lysates. However, although such assays may be sensitive, they are laborious, as cells must be lysed at individual time points. Furthermore, end point cGMP assays usually require the addition of PDE inhibitors to stop the breakdown of cGMP, leading to the accumulation and measurement of total cGMP, which can produce a type of “observational bias”, whereby the dynamics of cGMP are not easily detected, leading to an incomplete or misleading idea of the signalling patterns induced by different ligands [17]. Conversely, assays that can detect cGMP activity in real time will help remove the confounding effects of observational bias and will also allow more efficient examination of the kinetic aspects of signalling.

Genetically encoded biosensors based on Förster/fluorescence resonance energy transfer (FRET) are sometimes used to image cGMP activity in real time in single cells [18,19,20], where FRET refers to the transfer of energy from an excited donor fluorophore to an appropriate acceptor fluorophore when they are in close proximity [21]. These FRET-based biosensors for cGMP consist of a “sensor” domain that binds cGMP, which is sandwiched by an appropriate FRET donor/acceptor fluorophore pair such as cyan fluorescent protein (CFP)/yellow fluorescent protein (YFP). When cGMP binds to the sensor, the FRET pair moves either closer together or farther apart, producing a change in FRET that can be monitored in real time. However, although FRET-based biosensors are valuable for cellular microscopy and imaging of single cells with high spatial and temporal resolution, they are less suited for the detection of activity from populations of cells in plate-based assays, due to the higher background noise and photobleaching of the donor caused by laser excitation [22]. In contrast, bioluminescence resonance energy transfer (BRET), which uses a luciferase rather than a fluorescent protein as the donor, is a more sensitive technique for plate-based assays, as luciferases do not require laser excitation, but rather emit light after oxidation of a substrate molecule. As a result, BRET assays do not induce photobleaching of the donor, and show a lower background noise and a higher signal-to-noise ratio than FRET assays [22].

A BRET-based assay for cGMP activity would allow for high sensitivity, real-time detection of cGMP activity in live cells in a plate-based assay, which is more suited to ligand screening and pharmacological characterisation than imaging of single cells. Therefore, we report here the conversion of the cGES-DE5 FRET-based cGMP biosensor [19] to a BRET-based biosensor (cyclic GMP sensor using YFP-PDE5-Rluc8; CYGYEL), cloning of the novel biosensor into a lentiviral vector for stable expression in mammalian cells, validation and characterisation of the biosensor in live cells in a real-time multi-well plate-based format, and investigation of the applicability of the sensor for the detection of sGC- and pGC-mediated cGMP activity in human primary vascular cells.

## 2. Results

### 2.1. Design of a BRET-Based Biosensor for cGMP Activity and Generation of a Stably Expressing HEK293T Cell Line

**Figure 1:**
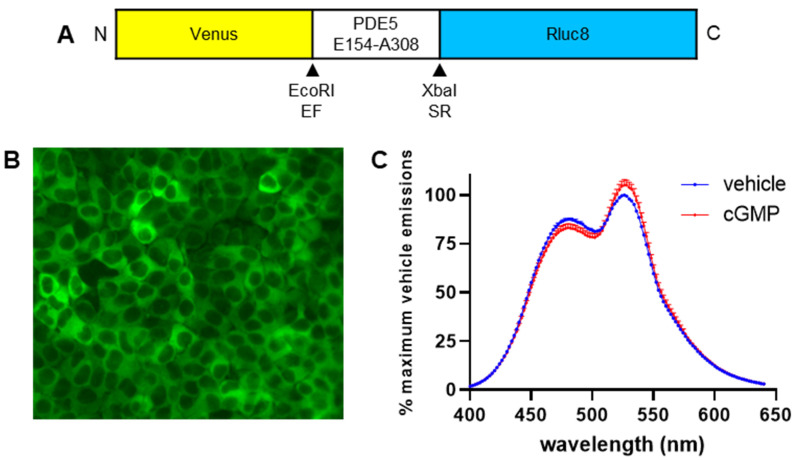
Design and expression of cyclic GMP sensor using YFP-PDE5-Rluc8 (CYGYEL), a bioluminescence resonance energy transfer (BRET)-based biosensor for cyclic guanosine monophosphate (cGMP) activity. (**A**) The isolated cGMP-binding domain from human phosphodiesterase (PDE) 5 is sandwiched between Venus and Rluc8. (**B**) HEK293T cells were transduced with CYGYEL lentivirus (HEK-CYGYEL cells) and sorted by fluorescence-activated cell sorting. A “high-expressing” population was imaged using a fluorescence microscope (20× magnification). (**C**) A spectral scan was generated after stimulating lysates of HEK-CYGYEL cells with cGMP (100 µM). Spectral scan data are the mean and standard error of the mean (SEM) of data from three independent assays.

Several FRET-based biosensors have been engineered and used to detect cGMP in single cells, including CGY [20], cygnet [18], and cGi [23], which use truncated forms of PKG as the “sensor” element, and cGES-DE5, which uses the cGMP-binding domain from human PDE5A as the “sensor” [19]. cGES-DE5 was chosen as the basis to generate a BRET-based biosensor due to its superior temporal resolution and high selectivity for cGMP over cAMP (approximately 420-fold), compared to other FRET-based sensors [19]. To convert cGES-DE5 to BRET, the sequence for the ECFP donor was replaced with the sequence for the luciferase Rluc8. Additionally, the sequence for EYFP was replaced with the sequence for Venus, due to its increased brightness (Figure 1A). The resulting sensor was named CYGYEL (cyclic GMP sensor using YFP-PDE5-Rluc8). Upon binding of cGMP to the cGMP-binding domain, the sensor was expected to respond by decreasing the distance between donor and acceptor, leading to an increase in BRET ratio.

As the biosensor was intended for use in human primary cells that are difficult to transfect, it was cloned into a lentiviral vector under the control of the constitutive human Ef1α promoter to enable viral delivery of the sensor. Lentivirus was produced and used to transduce HEK293T cells, which were sorted by FACS into populations that expressed low, medium, or high levels of the biosensor (Figure S2). To visualise the expression of CYGYEL in these cells, Venus emissions were detected from the “high” expressing cells using a GFP filter on a fluorescence microscope (Figure 1B), demonstrating that CYGYEL shows diffuse cytoplasmic expression, suggesting that it is likely to be able to detect cGMP throughout the cell in live cell assays. Furthermore, to determine whether CYGYEL responds to cGMP with an increase in BRET, the population of cells expressing “high” levels of CYGYEL were lysed, and spectral scans were generated from cell lysates in the absence or presence of cGMP (Figure 1C). As with cGES-DE5 [19], CYGYEL showed basal activation, but the relative intensity of emissions from Rluc8 relative to Venus changed upon the addition of cGMP (100 µM).

### 2.2. Characterisation of Real-Time cGMP Activity in Live Cells

**Figure 2:**
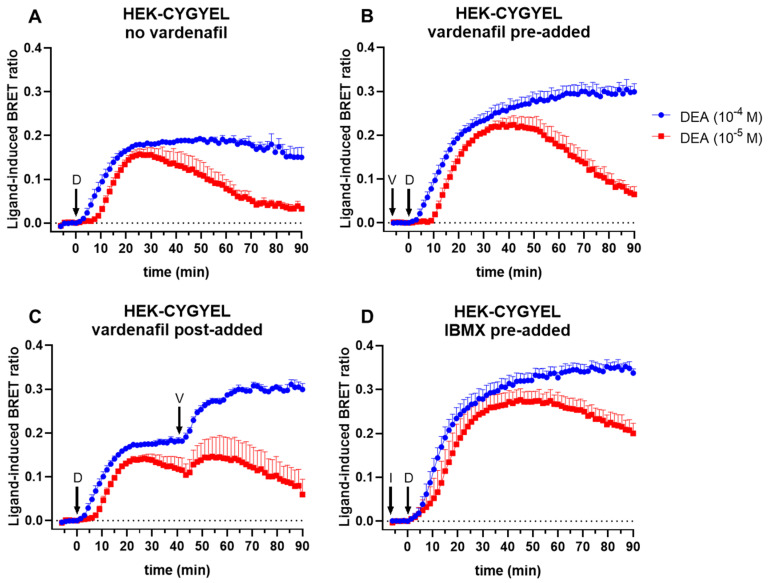
Characterisation of the CYGYEL BRET-based biosensor for cGMP activity in live cells. Intracellular cGMP was detected in real time after stimulation of HEK-CYGYEL cells with the nitric oxide (NO) donor DEA. Cells were stimulated with diethylamine NONOate (DEA) (D; 10 or 100 μM) for 90 min (min) either (**A**) without any PDE inhibitors, (**B**) with the cGMP-specific PDE5 inhibitor vardenafil (V; 100 nM) pre-added (approximately 10 min), (**C**) with vardenafil added after 40.5 min, or (**D**) with the nonselective PDE inhibitor 3-isobutyl-1-methylxanthine (IBMX) (I; 500 μM) pre-added (approximately 10 min). Time of ligand or vehicle addition is represented by arrows. Data are mean and SEM of three independent experiments.

CYGYEL was next characterised as a real-time signalling assay in live HEK293T cells. HEK-CYGYEL cells were stimulated for 90 min with the NO donor diethylamine NONOate (DEA), which releases NO via a pH-dependent mechanism [24], leading to the synthesis of cGMP via sGC (Figure 2). In the absence of PDE inhibitors, there were steep increases in the BRET ratio for at least 25 min following the treatment with DEA, representative of increases in cGMP (Figure 2A). The response then either remained relatively steady before showing a slow decline (DEA 100 μM) or declined for the duration of the time course (DEA 10 μM). As expected, preincubation with the cGMP-specific PDE5 inhibitor vardenafil (100 nM; approximately 10 min) led to more sustained increases in BRET ratio for both concentrations of DEA as well as higher maximum responses that peaked at approximately 40 min, which was much later than in the absence of vardenafil (Figure 2B), suggesting that the more transient responses seen without vardenafil were at least partly due to the PDE5-mediated degradation of cGMP. Similarly, the addition of vardenafil after an extended incubation with DEA (40.5 min) led to further increases in cGMP for both tested concentrations of DEA, although the effect was more pronounced for 100 μM DEA and was sustained for the duration of the time course, in contrast to 10 μM, which started to decline after a brief increase (Figure 2C). These results demonstrate that the biosensor is dynamic and able to detect robust increases and decreases in cGMP over an extended period of time (at least 90 min). To demonstrate the full dynamic range of the biosensor, cells were stimulated with DEA after pretreatment (approximately 10 min) with the nonselective PDE inhibitor IBMX (500 μM) (Figure 2D). In comparison to the response in the presence of vardenafil, the response for 10 μM DEA in the presence of IBMX was more sustained.

**Figure 3:**
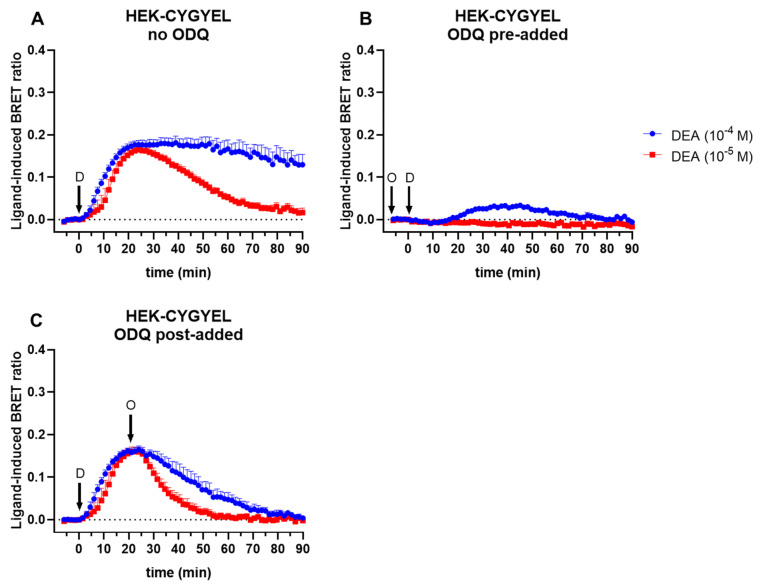
Blocking and reversing the cGMP sensor signal in HEK-CYGYEL cells. Intracellular cGMP was detected in real time after stimulation of HEK293T cells stably expressing CYGYEL with the NO donor DEA (D; 10 or 100 μM) in the absence or presence of the sGC inhibitor ODQ (O; 100 μM). (**A**) Cells were stimulated with DEA without ODQ. (**B**) Cells were stimulated with DEA after preincubation (approximately 10 min) with ODQ. (**C**) Cells were stimulated with DEA for 20.5 min before addition of ODQ. Time of ligand or vehicle addition is represented by arrows. Data are mean and SEM of three independent experiments.

To further demonstrate reversibility of the sensor, and blocking of the cGMP response, cells were stimulated with DEA in the absence or presence of the sGC inhibitor ODQ (100 μM) (Figure 3). The blockade of sGC with ODQ for approximately 10 min before addition of DEA either abolished the DEA response (10 μM DEA) or almost abolished it while also delaying the peak response until about 40 min rather than about 25 min (100 μM DEA) (Figure 3B). Furthermore, the addition of ODQ after a 20.5-min stimulation with DEA led to near-immediate decreases in the cGMP response (Figure 3C), again demonstrating that CYGYEL is specific for cGMP, dynamic, and reversible.

### 2.3. Selectivity of CYGYEL for cGMP over cAMP

**Figure 4:**
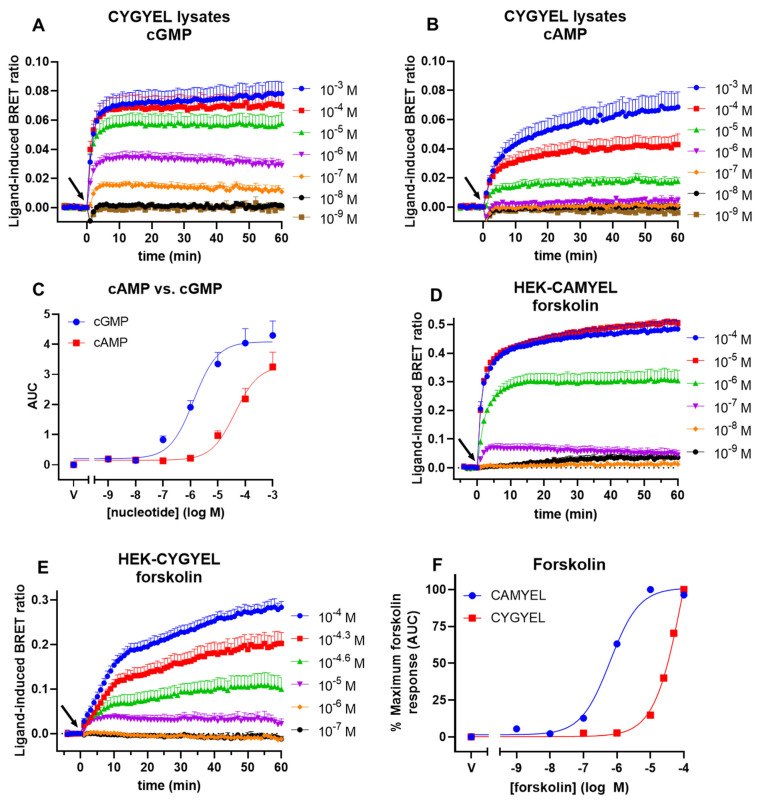
Activation of the CYGYEL cGMP sensor by cGMP and cAMP. Lysates from HEK293T cells stably expressing CYGYEL were stimulated with a range of concentrations of (**A**) cGMP or (**B**) cAMP for 60 min. (**C**) Concentration–response curves were generated by taking the area under the curve (AUC) from the time-course data. Live HEK293T cells stably expressing (**D**) the cAMP sensor using YFP-Epac-Rluc (CAMYEL) BRET-based cAMP biosensor or (**E**) CYGYEL were stimulated with a range of concentrations of forskolin for 60 min. (**F**) Concentration–response curves were generated by taking the area under the curve from the time-course data, normalised as a percentage of maximum forskolin response. Time of vehicle or ligand addition is represented by an arrow. Data are the mean and SEM of three or four independent experiments.

One concern for the use of genetically encoded biosensors for cGMP activity is that cAMP can still potentially bind to and “activate” the sensor, albeit with a lower potency than that of cGMP. This crosstalk might pose a problem when detecting signalling from ligands that increase the levels of both cGMP and cAMP, or when the basal concentration of cAMP inside the cell is high, leading to a high background activation of the sensor. The FRET-based biosensor cGES-DE5 was activated by cGMP with a potency of ~1.5 μM and by cAMP with a potency of ~630 μM, which corresponds to an approximately 420-fold higher selectivity for cGMP than cAMP [19]. To compare these results to our BRET variant, HEK-CYGYEL lysates were directly stimulated with a range of concentrations of cGMP or cAMP (Figure 4A–C). cGMP (Figure 4A) and cAMP (Figure 4B) both increased the BRET ratio in a concentration-dependent manner, however it was clear that higher concentrations of cAMP were required to activate the sensor relative to cGMP, and that cAMP also induced a slower activation of the sensor than cGMP, particularly at the higher concentrations of ligand (100 µM and 1 mM cAMP). Concentration–response curves were generated by taking the area under the curve, which revealed that cGMP activated CYGYEL with a potency of 1.32 ± 0.43 µM, whereas cAMP activated CYGYEL with a potency of 42.15 ± 15.44 µM (Figure 4C; Table 1). Thus, cGMP showed an approximately 32-fold higher selectivity for activation of CYGYEL relative to cAMP, compared with the higher selectivity seen for cGES-DE5.

However, although these results reflect the biochemistry that occurs in cell lysates, they do not necessarily reflect what may occur in pharmacological assays using live cells, as cell lysate assays do not reflect, for example, the localisation of second messengers into microdomains or localised PDE activity. Thus, to understand whether cAMP activates CYGYEL in live cell assays, we compared cAMP-mediated activation of CYGYEL to cAMP-mediated activation of the cAMP sensor CAMYEL [25] in live cells by stimulating HEK-CYGYEL and HEK-CAMYEL cells with the adenylate cyclase activator forskolin (Figure 4D–F). Time courses showed sustained increases in BRET ratio for the duration of a 60 min stimulation for both CAMYEL (Figure 4D) and CYGYEL (Figure 4E). Concentration–response curves revealed that the forskolin response was detected with a potency of 0.62 ± 0.06 μM in CAMYEL cells and 100.5 ± 11.8 μM in CYGYEL cells (Figure 4F; Table 2). Thus, in live HEK293T cells, cAMP activated CAMYEL with a 160-fold higher potency than it activated CYGYEL.

### 2.4. Detection of cGMP in Human Primary Vascular Cells

We next aimed to determine whether CYGYEL can effectively detect cGMP activity in human primary cells. Given that cGMP is a key signalling pathway for the regulation of cardiovascular physiology, we transduced human primary vascular cells, specifically, human umbilical vein endothelial cells (HUVECs) and human umbilical vein smooth muscle cells (HUVSMCs), with CYGYEL. Fluorescence-activated cell sorting was then used to remove cells that were not successfully transduced (Figures S3 and S4).

**Figure 5:**
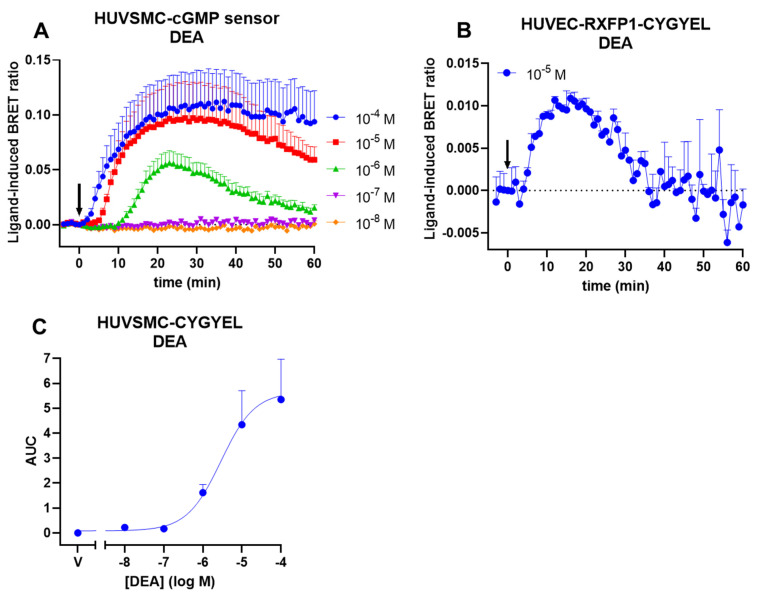
DEA-induced cGMP activity in human umbilical vein smooth muscle cells (HUVSMCs) and human umbilical vein endothelial cells (HUVECs). HUVSMCs and HUVECs stably expressing CYGYEL were stimulated with vehicle or the nitric oxide donor DEA for 60 min. Time courses were generated for (**A**) HUVSMCs and (**B**) HUVECs. (**C**) Concentration–responses curves were generated by taking the area under the curve (AUC) from the time-course data. Time of vehicle or DEA addition is represented by an arrow. Data are the mean and SEM for two (panel B) or four independent experiments.

To detect sGC-mediated cGMP activity, HUVSMC-CYGYEL and HUVEC-RXFP1-CYGYEL cells were stimulated with the NO donor DEA, in the absence of any PDE inhibitors (Figure 5). Time courses for HUVSMCs showed robust, concentration-dependent increases in cGMP activity (Figure 5A), which were transient at submaximal concentrations of DEA. In contrast, concentration-dependent data were unable to be generated for HUVECs, as responses were difficult to detect (Figure 5B). Concentration–response curves were generated for HUVSMCs, demonstrating a potency (EC_50_) in the micromolar range (Figure 5C, Table 3).

**Figure 6:**
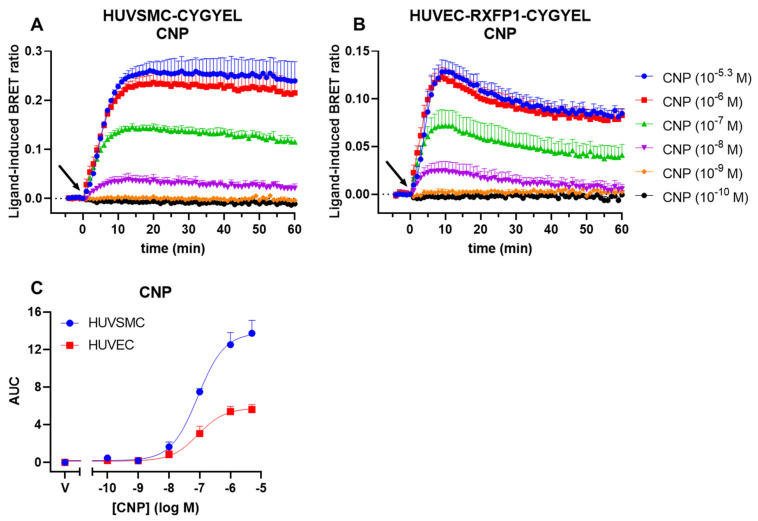
CNP-induced cGMP activity in HUVSMCs and HUVECs. HUVSMCs and HUVECs stably expressing CYGYEL were stimulated with vehicle or C-type natriuretic peptide (CNP) for 60 min. Time courses were generated for (**A**) HUVSMCs and (**B**) HUVECs. (**C**) Concentration–response curves were generated by taking the AUC from the time-course data. Time of vehicle or CNP addition is represented by an arrow. Data are the mean and SEM for three independent experiments.

In addition to sGC-mediated cGMP production, CYGYEL was tested for its ability to detect pGC-mediated cGMP activity in human vascular cells (Figure 6). HUVSMCs and HUVECs were stimulated with a range of concentrations of C-type natriuretic peptide (CNP), which activates the GC-B form of pGC, for 60 min. Time courses generated for HUVSMCs (Figure 6A) and HUVECs (Figure 6B) showed concentration-dependent increases in cGMP activity after CNP stimulation, which reached maximum activity at 1–5 μM for both cell types. However, whereas the cGMP responses in HUVSMCs were relatively sustained for the duration of the time course, the cGMP responses in HUVECs peaked at about 10 min before showing a slow decline that started to stabilise by the end of the time course. Concentration–response curves demonstrated that CNP had a near-identical potency for cGMP increases in both cell types (Figure 6C, Table 3). Additionally, the shapes of the time courses were different for CNP compared with DEA, with CNP responses being more sustained, consistent with the different mechanisms of action of these two different types of drugs.

## 3. Discussion

Traditional end point signalling assays for cGMP do not easily measure the temporal aspects of signalling as they require lysing cells at individual time points and usually employ PDE inhibitors to allow the accumulation of cGMP to detectable levels. The ability of BRET-based biosensors to detect signalling in real time in plate-based cellular assays prompted us to develop a BRET-based biosensor for cGMP activity. The FRET-based biosensor cGES-DE5 was converted to BRET due to its high selectivity for cGMP over cAMP and high temporal resolution compared with other available biosensors [19]. This was accomplished by substituting the region coding for ECFP with Rluc8, as well as swapping the region coding for EYFP to Venus. The resulting biosensor, which was named CYGYEL, was cloned into a lentiviral vector and subsequently tested across several cell types including human primary vascular cells, where it successfully detected real-time changes in cGMP activity, and in some cases showed differences in temporal patterns between ligands and cell types.

First, HEK293T cells were transduced and sorted by FACS for basic characterisation of the sensor. The imaging of HEK-CYGYEL cells using a fluorescence microscope demonstrated that CYGYEL was expressed throughout the cytoplasm, suggesting that the detection of cGMP throughout the cell would be possible. Real-time assays in these cells showed that CYGYEL detects robust increases and decreases in sGC-mediated cGMP activity for extended periods of time (at least 90 min) after stimulation with a NO donor. The assay was sensitive enough to detect cGMP activity in the absence of any PDE inhibitors, but the addition of the cGMP-specific PDE5 inhibitor vardenafil or the nonspecific PDE inhibitor IBMX did increase the BRET ratio and prolong the response. Furthermore, preincubation with the sGC inhibitor ODQ diminished or abolished the increases in BRET ratio, depending on the DEA concentration, further confirming the assay was specific for cGMP activity.

Following this, we demonstrated that CYGYEL can detect cGMP activity mediated through both sGC and pGC in human primary vascular cells, specifically HUVECs and HUVSMCs. Interestingly, we observed differences in cGMP signalling both between cell types and between ligands. Both cell types showed robust, concentration-dependent responses to CNP, but cGMP activity was more sustained in HUVSMCs than in HUVECs. Notably, however, the potency of CNP was almost identical between cell types. In contrast, DEA responses were weak and difficult to detect in HUVECs, whereas there were robust, concentration-dependent DEA-mediated cGMP increases in HUVSMCs, which were also substantially less potent than the CNP responses. Furthermore, CNP showed faster increases in cGMP compared with DEA, consistent with the role of CNP as a membrane receptor agonist, compared with DEA, which is a NO donor. Additionally, the weak responses to DEA observed in HUVECs, compared with strong responses to CNP observed in the same cell type, are consistent with findings from other studies showing that natriuretic peptides induced stronger cGMP responses in HUVECs compared with NO donors [26,27]. Finally, as robust cGMP activity was detected in both types of vascular cells tested, it is expected that the lentiviral vector, which uses a constitutive human promoter, will also aid in the detection of cGMP across a variety of other cell types.

One potential concern for the use of genetically encoded cGMP sensors is that cAMP can also bind to cGMP-binding domains, including the binding domain from PDE5, albeit with a lower affinity than cGMP. As noted, the low crosstalk from cAMP observed for cGES-DE5 was one of the primary reasons this sensor was chosen as the basis to generate CYGYEL. In lysates from TsA201 cells, cGES-DE5 was activated by cGMP with a potency of about 1.5 μM and by cAMP with a potency of about 630 μM [19]. Here, we demonstrated that CYGYEL was activated by cGMP with a potency (EC_50_) of about 1.3 μM and by cAMP with a potency of about 42 μM—a selectivity of about 32-fold. Thus, although the EC_50_ of cGMP is comparable between the two variants of the biosensor, the EC_50_ of cAMP is considerably lower for the activation of CYGYEL than cGES-DE5. As noted, however, results from such biochemical assays in cell lysates will not always reflect what occurs in live cells due to a variety of factors such as localised cyclic nucleotide and PDE activity. Therefore, we also tested the ability of cAMP to activate CYGYEL in live cell assays by stimulating HEK-CYGYEL cells with the adenylate cyclase activator forskolin and compared these results to those observed for the same assay conducted in HEK-CAMYEL cells. These findings showed that in contrast to the activation of CAMYEL, relatively high concentrations of forskolin were required to induce a robust activation of CYGYEL, and that there was a 160-fold difference in forskolin potencies between the activation of the two sensors. However, it should also be noted that cAMP-mediated activation of CYGYEL may depend on the cell type being tested due to differential expression of adenylate cyclases and PDEs, for example, so users of this biosensor should carefully consider this. On the other hand, it is also important to note that for many applications of CYGYEL, such as screening of NO donors and pGC agonists as conducted here, the crosstalk from cAMP may not be an important factor. Examples of situations where crosstalk may be important include situations when using a ligand induces the activation of both cGMP and cAMP, or when using cell types that have high background levels of cAMP. Furthermore, other characteristics of a sensor, such as its temporal resolution, are also equally important to consider in the choice of a biosensor. As noted, for example, the superior temporal resolution of cGES-DE5 reported by Nikolaev et al. [19], compared with other available FRET-based biosensors, was the other key reason that we chose cGES-DE5 as the basis for our BRET variant.

We also note the development of another BRET-based biosensor for cGMP, known as GFP^2^-GAFa-Rluc [28]. This biosensor is based on the BRET^2^ methodology [29], using the combination of Rluc, GFP^2^, and DeepBlueC as the substrate, compared with CYGYEL which is based on BRET^1^, using the combination of Rluc8, Venus, and coelenterazine *h*. GFP^2^-GAFa-Rluc uses a longer segment of PDE5A than CYGYEL, which is sandwiched between the Rluc donor and GFP^2^ acceptor. In lysates from transfected cells, cGMP had a surprisingly low EC_50_ of approximately 30 nM for the activation of GFP^2^-GAFa-Rluc, and cAMP did not appear to activate the sensor, although it was only tested up to 1 µM in lysates [28]. This finding is intriguing as it raises the possibility that this truncation of PDE5 may be more optimal than the one described by Nikolaev et al. [19], which was incorporated into CYGYEL. However, two caveats must be noted. First, GFP^2^-GAFa-Rluc may act as a “sink” that sequesters cGMP in the cell, suggesting that cGMP may have a slow off-rate and remains bound, preventing its degradation by PDEs, thus making it difficult to detect the kinetics of signalling [28]. Second, our own results have demonstrated that CYGYEL is dynamic and reversible, able to detect the kinetics of cGMP activity over extended periods of time; yet similar kinetic assays were not conducted using GFP^2^-GAFa-Rluc [28]. This may be in part related to the use of the BRET^2^ methodology, which does not typically allow extended time courses due to weak emissions and a rapid loss of signal [30]. In contrast, BRET^1^ maintains a high luminescence for a longer duration and allows for a higher throughput. Due to these factors, we cannot directly compare the activity of the two biosensors and are unsure of the temporal resolution of GFP^2^-GAFa-Rluc. Future studies could convert GFP^2^-GAFa-Rluc to BRET^1^ in order to directly compare the temporal resolution and reversibility of the two different truncations of PDE5, in order to determine the utility of each truncation as a sensor domain.

Similarly, future studies could also aim to increase the affinity of cGMP for CYGYEL or other cGMP biosensors while at the same time decreasing the affinity of cAMP, with the caveat that very high affinities might lead to sequestration cGMP [28]. Interestingly, a variant of cGES-DE5 that swapped the ECFP/EYFP FRET pair to a T-sapphire/dimer2 FRET pair showed substantial increases in both affinity for cGMP (~40 nM) and selectivity for cGMP over cAMP [31], allowing the detection of cGMP in adult cardiomyocytes [32]. However, as we have shown, changing the ECFP/EYFP pair to Rluc8/Venus did not have the same effect on selectivity. Additionally, the recently developed FRET-based cGMP sensor *Pf*PKG, which was based on PKG (rather than PDE5) from *Plasmodium falciparum*, showed a high cGMP affinity of about 23 nM, but this was coupled with a high cAMP affinity of about 4.6 µM, suggesting that it may be difficult to decouple the affinities of the two cyclic nucleotides for cGMP-binding domains [33]. In fact, the authors even mutated the cGMP-binding domain from PKG in an attempt to reduce the affinity of cAMP, but were unsuccessful [33].

The compartmentalisation of cyclic nucleotides is a current research topic in cell biology [34], and although cGMP compartmentalisation is less well understood than that of cAMP, it does appear that there are spatially segregated pools of cGMP within the cell [35,36], which is not surprising given that sGC and pGC are already separately localised. Importantly, microdomains of cGMP may have different functional roles in cardiovascular physiology, which is still under investigation [37]. The ability of a biosensor to detect cGMP, and its selectivity for cGMP over cAMP, may differ depending on whether lysates or live cells are stimulated, and may also differ based on cell type, due to the differential expression and compartmentalisation of enzymes that regulate cyclic nucleotide levels within the cell. Notably, CYGYEL showed diffuse cytoplasmic expression when cells expressing CYGYEL were imaged using a fluorescence microscope, which was consistent with its ability to detect cGMP synthesised by either sGC or pGC in live cells. Although BRET is not typically used to detect localised signalling, it may be possible to do this by targeting the biosensor to particular locations of interest, such as the membrane [38]. Alternatively, cGES-DE5 may be used as a complementary biosensor to detect localised signalling via sGC versus pGC in single cells, for example, which is one of the primary advantages of FRET over BRET. Additionally, the recent development of enhanced Nano-lanterns [39] enabled the generation of a dual FRET/BRET-based cAMP biosensor [40], and it would be interesting to see whether a similar approach is viable for the generation of a single cGMP sensor that can be used for plate-based assays in addition to cellular microscopy.

In summary, we have generated a genetically encoded, BRET-based biosensor for cGMP activity that streamlines the process of detecting cGMP compared to traditional end point assays. CYGYEL can detect changes in sGC- and pGC-mediated cGMP activity over time in the plate-based format, and the lentiviral vector allows for the generation of human cell lines stably expressing the biosensor, allowing the detection of cGMP across physiologically relevant cell types.

## 4. Methods

### 4.1. Materials and Reagents

1H-[1,2,4]Oxadiazolo[4,3-a]quinoxaline-1-one (ODQ), 3-isobutyl-1-methylxanthine (IBMX), adenosine 3′,5′-cyclic monophosphate sodium salt monohydrate, C-type natriuretic peptide (CNP), diethylamine NONOate diethylammonium salt (DEA), forskolin, guanosine 3′,5′-cyclic monophosphate sodium salt, Polybrene, Tris base, and vardenafil, were from Sigma-Aldrich (Burlington, MA, USA). Dulbecco’s modified Eagle medium (DMEM), Medium 199 (M199), L-glutamine, and penicillin–streptomycin (P/S) were from Gibco (Waltham, MA, USA). Foetal bovine serum (FBS) was from Scientifix (Melbourne, VIC, Australia). EGM-2 endothelial cell growth medium-2 BulletKit was from Lonza (Basel, Switzerland). Smooth muscle cell growth supplement was from ScienCell (Carlsbad, CA, USA). Ethylenediaminetetraacetic acid disodium salt dihydrate (EDTA) was from ChemSupply (Adelaide, SA, Australia). Coelenterazine *h* was from Nanolight (Pinetop, AZ, USA).

### 4.2. Design of a BRET-Based Biosensor for cGMP Activity

The BRET-based biosensor CYGYEL was created based on the cGES-DE5 FRET-based biosensor [19]. cGES-DE5 consists of the cGMP-binding domain (Glu154 to Ala308) from human PDE5A1 (GenBank accession number NM_001083) inserted between enhanced yellow fluorescent protein (EYFP) and enhanced cyan fluorescent protein (ECFP) using the *Eco*RI and *Xba*I restriction sites, respectively. The sequence for EYFP was replaced with the sequence for Venus, and the sequence for ECFP was replaced with the sequence for Rluc8. A Kozak sequence (GCCACC) was placed before the start codon, and the gene for the biosensor was synthesised by GenScript (Piscataway, NJ, USA) into pcDNA3.1/Zeo^(+)^ between the *Nhe*I and *Apa*I restriction sites.

### 4.3. Cell Culture

All cells were cultured at 37 °C with 5% CO_2_. HEK293T cells (CRL-3216) were cultured in DMEM supplemented with 10% FBS, 1% L-glutamine, and 1% P/S. Human umbilical vein smooth muscle cells (HUVSMCs) were grown in M199 supplemented with 5% heat-inactivated FBS, 1% P/S, and smooth muscle cell growth supplement. Human umbilical vein endothelial cells (HUVECs) were grown in EGM-2 endothelial cell growth medium-2 (including 2% FBS) supplemented with 1% P/S. These are henceforth referred to as “complete media”. HUVSMCs were purchased from ScienCell.

### 4.4. Isolation of Human Umbilical Vein Endothelial Cells

This study was approved by the Monash Health Human Research and Ethics Committee (HREC: 01067B). Healthy term umbilical cord from singleton pregnancies were collected, with informed written consent, at the time of elective caesarean section. Exclusion criteria included multiple pregnancy, maternal medical conditions, any history of preeclampsia and/or foetal growth restriction, smoking, alcohol or drug use during pregnancy. Exclusions were made for the following medications: antihypertensive, aspirin, nonsteroidal anti-inflammatory drugs, or thyroid medications. To isolate HUVECs, the vein of the umbilical cord (~15 cm) was rinsed with 1× Hank’s Buffered Saline Solution (HBSS) until all blood was removed. Luer-lok fittings were placed at both ends of the vein and 10 mL of collagenase type II (0.5 mg/mL in HBSS) syringed into the vein. The cord was then incubated for 10 min at 37 °C in a humidified 95% air/5% CO_2_ incubator. The collagenase was collected by a syringe and placed in a 50 mL falcon tube. The vein was washed twice with 10 mL EBM^TM^-2 endothelial cell growth media (Lonza), which was collected and added into the falcon tube. The tube was spun at 500× *g* for 5 min at room temperature to pellet the cells. Cells were resuspended in 5 mL EBM^TM^-2 media and cultured in a T25 flask at 37 °C in a humidified incubator at 95% air/5% CO_2_. Cells were passaged to P2 before slowly freezing in 90% FBS and 10% DMSO (1 million cells/mL) and stored in liquid nitrogen.

### 4.5. Cloning CYGYEL into a Lentiviral Vector

CYGYEL was cloned into a lentiviral vector under the control of the constitutive human Ef1α promoter using Gateway technology (Invitrogen), according to the manufacturer’s instructions. The CYGYEL coding sequence from the pcDNA3.1/Zeo^(+)^ vector was flanked with appropriate *att*B5 and *att*B2 sequences and amplified by polymerase chain reaction (PCR) using the forward primer 5′ GGGG ACA ACT TTG TAT ACA AAA GTT GGC CAC CAT GGT GAG CAA GGG C 3′ and reverse primer 5′ GGGG ACC ACT TTG TAC AAG AAA GCT GGG TAT TA CTG CTC GTT CTT CAG CAC 3′. The PCR product of the correct size was extracted, gel-purified, and cloned into the pDONR 221 P5-P2 vector using BP Clonase II. The resulting pENTR L5-L2 entry clone was confirmed to have the correct sequence using Sanger sequencing. Our pENTR L1-R5 Ef1α entry clone was cloned along with the pENTR L5-L2 CYGYEL clone into the pLenti X1 Zeo DEST vector [41] using LR Clonase II, to generate the pLenti X1 Ef1α CYGYEL expression clone.

### 4.6. Generation of HEK-CAMYEL and HEK-CYGYEL Cell Lines via Lentiviral Transduction

HEK293T stably expressing the cAMP sensor CAMYEL [25] or CYGYEL were generated using crude lentivirus. HEK293T cells were plated at 3.5 million cells on a 10 cm dish. The next day, cells were transfected with 8 µg of either pLenti X1 Ef1α CAMYEL [42] or pLenti X1 Ef1α CYGYEL, along with the lentiviral packaging and envelope plasmids pMDL, pRSV-Rev, and pCMV-VSV-G, using Lipofectamine 2000. The following day, HEK293T cells to be transduced were plated on a 10 cm dish at 400,000 cells. The next day, media containing lentivirus from transfected cells were harvested and filtered through a 0.45 µm syringe filter. The newly plated HEK293T cells were transduced by replacing the cell culture media with the media containing lentivirus, mixed with 4 μg/mL hexadimethrine bromide (Polybrene) to increase transduction efficiency. Three rounds of transduction were performed over 32 h, before allowing cells to recover for three days in complete DMEM.

### 4.7. Purification of Lentivirus and Transduction of Primary Vascular Cells

CYGYEL lentivirus was produced in HEK293T cells and purified for transduction of primary cells. HEK293T cells were plated out on five 20 cm dishes at 11 million cells per dish. The next day, cells were transfected with the pLenti X1 Ef1α CYGYEL expression clone, pMDL/pRRE, pRSV-Rev, and pCMV-VSV-G, using Lipofectamine 2000. Two days later, media containing secreted lentivirus was harvested and spun at 2000 rpm for five minutes to pellet any cellular debris. The supernatant was then filtered through a 0.45 µm filter and spun in a Hitachi CP100MX ultracentrifuge at approximately 72,000× *g* at 4 °C for two and a half hours to pellet the virus. The supernatant was then discarded, and 50 µL of cold phosphate-buffered saline (PBS) was added per tube (for a total of 150 µL per viral preparation), which was stored at 4 °C overnight. Some of the crude lentivirus-containing media were also stored at 4 °C overnight. The next day, the virus was resuspended in the PBS, pooled, then stored in aliquots at −80 °C. Moreover, 1 µL of the virus was also used for a visual titration on HEK293T cells, along with some of the crude lentivirus, to ensure that it was of high enough titre to transduce cells in vitro.

HUVECs and HUVSMCs were transduced with the purified virus in a T175 flask at approximately 70% confluency. Then, 5 μL of purified lentivirus was resuspended in cell culture media along with 4 μg/mL hexadimethrine bromide (Polybrene). Cell culture media in the flask were replaced with virus-containing media, which were left on cells overnight before replacing it with fresh cell culture media.

### 4.8. Flow Cytometry and Fluorescence-Activated Cell Sorting

Transduced cells were sorted from non-transduced cells using fluorescence-activated cell sorting (FACS), using a Becton Dickinson FACSAria III. Cells were isolated by gating after plotting the forward scatter area (FSC-A) against the side scatter area. Single cells were isolated after plotting the side scatter height against the side scatter width and the forward scatter height (FSC-H) against the forward scatter width. Dead cells stained with DAPI were removed after laser excitation at 405 nm and gating FSC-A against emissions at 450/40 nm. From the population of single, live cells, cells showing higher YFP emissions than control cells, as detected after laser excitation at 488 nm and emissions at 530/30 nm, were sorted into separate populations, as noted. Cells were subsequently grown to confluency in a T175 flask and cryopreserved. For HUVECs, the cells were already expressing “high” levels of a relaxin family peptide receptor 1 (RXFP1)–internal ribosome entry site (IRES)–green fluorescent protein (GFP) construct, from a previous transduction and sort (Figure S1); however, emissions were separated from YFP emissions by using separate emission filters for GFP (510/20 nm) and YFP (530/30 nm) and applying compensation. These cells had subsequently been cryopreserved. For analytical flow cytometry, cells were isolated as above, but with the following exceptions: a Beckman Coulter CytoFLEX S was used, single cells were isolated after plotting only for FSC-A versus FSC-H, and YFP was detected using a 525/40 nm emission filter. 

### 4.9. Fluorescence Microscopy

HEK293T cells that were stably expressing “high” levels of CYGYEL were imaged using a Leica DM IL LED microscope with DFC450 C camera, EL6000 external light source, and GFP filter, at 20× magnification.

### 4.10. BRET Assays in Live Cells

HEK293T cells, HUVSMCs, or HUVECs stably expressing either CYGYEL or CAMYEL were seeded onto 96-well CulturPlate microplates (PerkinElmer, Waltham, MA, USA) and were incubated overnight. At the time of the assay, HEK293T or HUVSMC cell culture media were replaced with phenol red free complete media containing 25 mM HEPES and 5 µM coelenterazine *h*, whereas HUVEC cell culture media, EGM-2, were replaced with complete EGM-2 containing 25 mM HEPES and 5 µM coelenterazine *h*. Emissions were detected simultaneously from Rluc (CAMYEL) or Rluc8 (CYGYEL) (475/30 nm) and citrine (CAMYEL) or Venus (CYGYEL) (525/30 nm) using a PHERAstar *FSX* plate reader (BMG Labtech, Ortenberg, Germany) at 37 °C. Emissions were read for five minutes to establish a baseline before addition of a vehicle or ligand.

### 4.11. BRET Assays in Cell Lysates

HEK-CYGYEL cells were washed and resuspended in DPBS, and then centrifuged in order to pellet the cells. DPBS was aspirated and cells were resuspended in buffer containing 5 mM Tris and 2 mM EDTA (pH 7.3), then sonicated on low power for three 10 s cycles at 4 °C. Lysates were then spun down at 21,000× *g* for 20 min at 4 °C, after which the supernatant was obtained and stored at −80 °C or used for BRET assays. During the assay, lysates were mixed with coelenterazine *h* (5 µM), distributed into a white, 384-well OptiPlate (PerkinElmer) with lysates corresponding to the equivalent of approximately 5000 cells per well, and then assayed as above using a PHERAstar *FSX* plate reader. Cell lysates were also used to generate spectral scans, using a CLARIOstar plate reader (BMG Labtech).

### 4.12. Presentation of BRET Data

The ligand-induced BRET ratio was calculated by subtracting the ratio of acceptor channel emissions (525/30 nm) to donor channel emissions (475/30 nm) for vehicle wells from the same ratio for treated wells:

$$\mathrm{ligand}\text{-}\mathrm{induced}\;\mathrm{BRET}\;\mathrm{ratio}=\frac{\mathrm{acceptor}\;\mathrm{channel}\;(\mathrm{ligand})}{\mathrm{donor}\;\mathrm{channel}\;(\mathrm{ligand})}-\frac{\mathrm{acceptor}\;\mathrm{channel}\;(\mathrm{vehicle})}{\mathrm{donor}\;\mathrm{channel}\;(\mathrm{vehicle})}$$

For time courses, the ligand-induced BRET ratio was plotted against time, with the last prereading before ligand addition displayed as the zero time point (time of vehicle/ligand addition). For CAMYEL cAMP data only, decreases in ligand-induced BRET ratio represent increases in cAMP, so all values were subtracted from zero. For spectral scan data, the wavelength (nm) was plotted against emissions. Concentration–response curves were fit to the data by applying three-parameter nonlinear regressions to the area under the curve data, using Prism 9 from GraphPad (San Diego, CA, USA).

## Figures and Tables

**Table 1:** Potency of cGMP for activation of CYGYEL. HEK293T cells stably expressing CYGYEL were lysed and stimulated with a range of concentrations of cGMP or cAMP. Potencies were calculated by taking the area under the curve from the time-course data. Selectivity was determined by dividing the EC_50_ for cAMP by the EC_50_ for cGMP. Data are the mean and SEM of four independent experiments. * *p* < 0.05 compared to cGMP in an unpaired *t*-test.

	cGMP	cAMP	cAMP/cGMP
**EC_50_ (µM)**	1.32 ± 0.43	42.15 ± 15.44	32
**pEC_50_**	5.88 ± 0.14	4.38 ± 0.16 *	

**Table 2:** Forskolin-mediated cAMP activation of the CAMYEL and CYGYEL biosensors expressed in HEK293T cells. HEK293T cells stably expressed with either the CAMYEL cAMP sensor or CYGYEL were stimulated with a range of concentrations of forskolin. Concentration–response curves were generated by taking the area under the curve from the time-course data, normalised to maximum forskolin response in order to compare the potencies (EC_50_ and pEC_50_) of forskolin for downstream activation of each biosensor. Data are mean and SEM of three independent experiments. * *p* < 0.05 compared to HEK-CAMYEL in an unpaired *t*-test.

Forskolin	HEK-CAMYEL	HEK-CYGYEL
**EC_50_ (µM)**	0.62 ± 0.06	100.5 ± 11.8
**pEC_50_**	6.21 ± 0.04	3.99 ± 0.05 *

**Table 3:** Potency of DEA and CNP for cGMP activity in HUVSMCs and HUVECs. HUVSMCs and HUVECs stably expressing CYGYEL were stimulated with the NO donor DEA or the GC-B agonist CNP for 60 min. The potencies (EC_50_ and pEC_50_) were determined by taking the area under the curve (AUC) from the time-course data. Data are the mean and SEM for three or four independent experiments.

	DEA		CNP	
	HUVSMC	HUVEC	HUVSMC	HUVEC
**EC_50_**	2.88 ± 2.34 µM	–	87.01 ± 20.39 nM	88.97 ± 29.41 nM
**pEC_50_**	5.54 ± 0.35	–	7.06 ± 0.10	7.05 ± 0.14

## Data Availability

Not applicable.

## Supplementary Materials

The following supporting information can be downloaded at: https://www.mdpi.com/article/10.3390/ijms23031908/s1.
