# Supplementary material for: A Real-Time, Plate-Based BRET Assay for Detection of cGMP in Primary Cells
Source: Int J Mol Sci. 2022 Feb 8;23(3):1908. doi: 10.3390/ijms23031908 (PMC8837005; doi:10.3390/ijms23031908)
Supplement: Supplementary file 1 [file ijms-23-01908-s001.zip › ijms-1533084-supplementary.pdf]

Supplementary figures

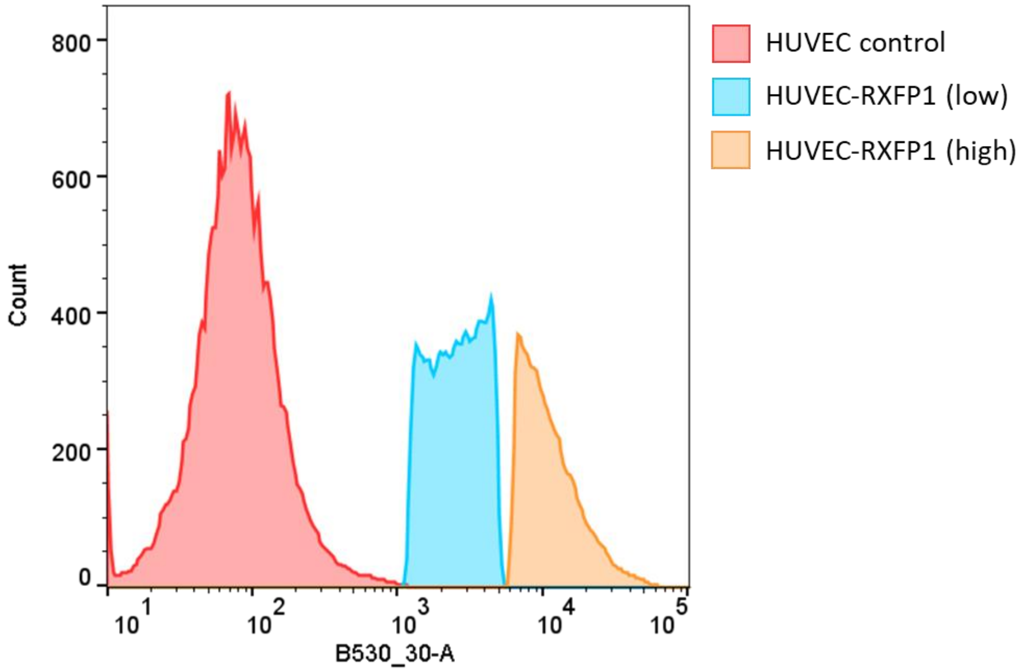

Figure S1. Representation of fluorescence-activated cell sorting of HUVEC-RXFP1 cells. Human umbilical vein endothelial cells (HUVECs) were transduced with relaxin family peptide receptor 1 (RXFP1)–internal ribosome entry site (IRES)–green fluorescent protein (GFP) lentivirus and then sorted by fluorescence-activated cell sorting (FACS) to remove non-transduced cells and to sort transduced cells into "low" (blue) and "high" (orange) populations. Single, live cells were isolated by gating, and cells that expressed RXFP1 (based on GFP emissions that were higher than control cells) were sorted into separate populations (excitation: 488 nm; emission 530/30 nm). Non-transduced control cells are shown in red.

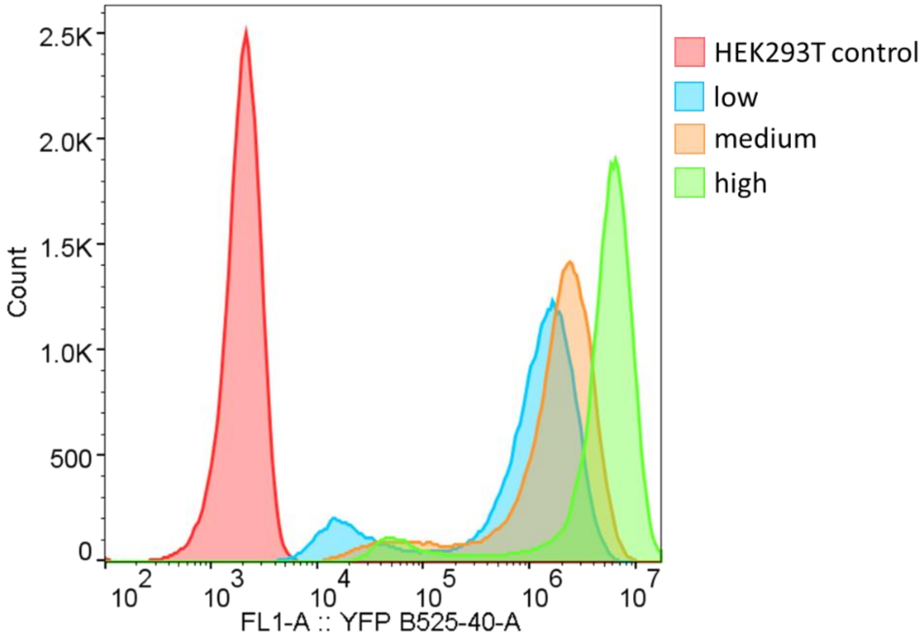

Figure S2. Analytical flow cytometry of HEK-CYGYEL stable cells. HEK293T cells were transduced with CYGYEL lentivirus and were sorted into populations expressing "low", "medium", or "high" levels of the sensor. Cells were later checked for YFP expression using analytical flow cytometry. Single, live cells were isolated by gating, after which histograms showing mean YFP emissions were generated (excitation: 488 nm; emission: 525/40 nm).

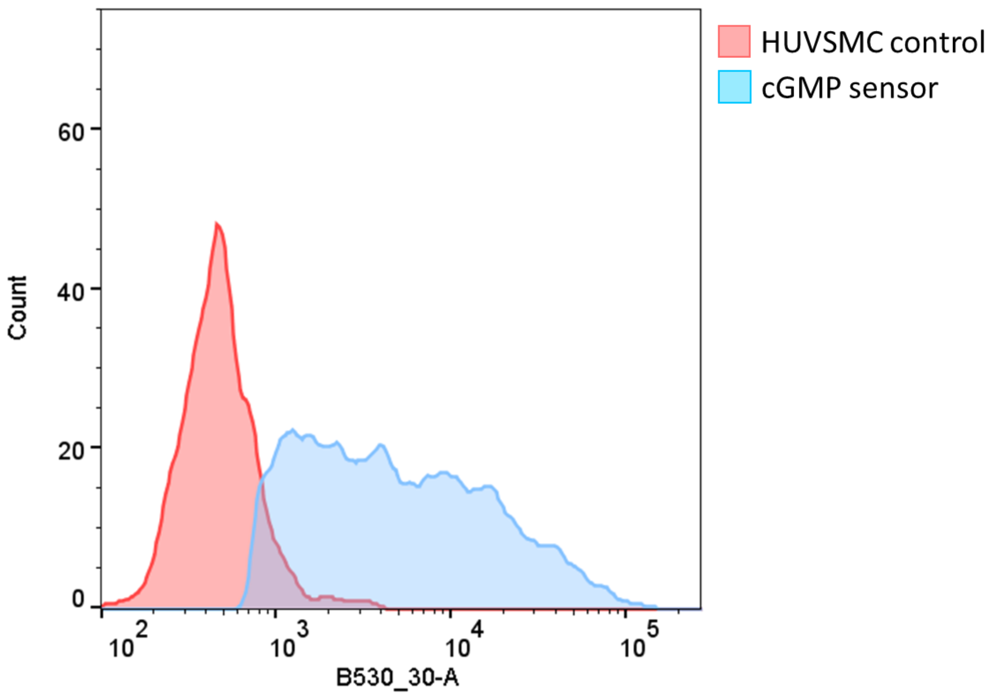

Figure S3. Representation of fluorescence-activated cell sorting (FACS) for HUVSMC-CYGYEL cells. HUVSMCs were transduced with CYGYEL lentivirus and then sorted by FACS to remove non-transduced cells. Single, live cells were isolated by gating, and cells that expressed CYGYEL (based on YFP emissions that were higher than control cells) were sorted into a separate population (excitation: 488 nm; emission: 530/30 nm).

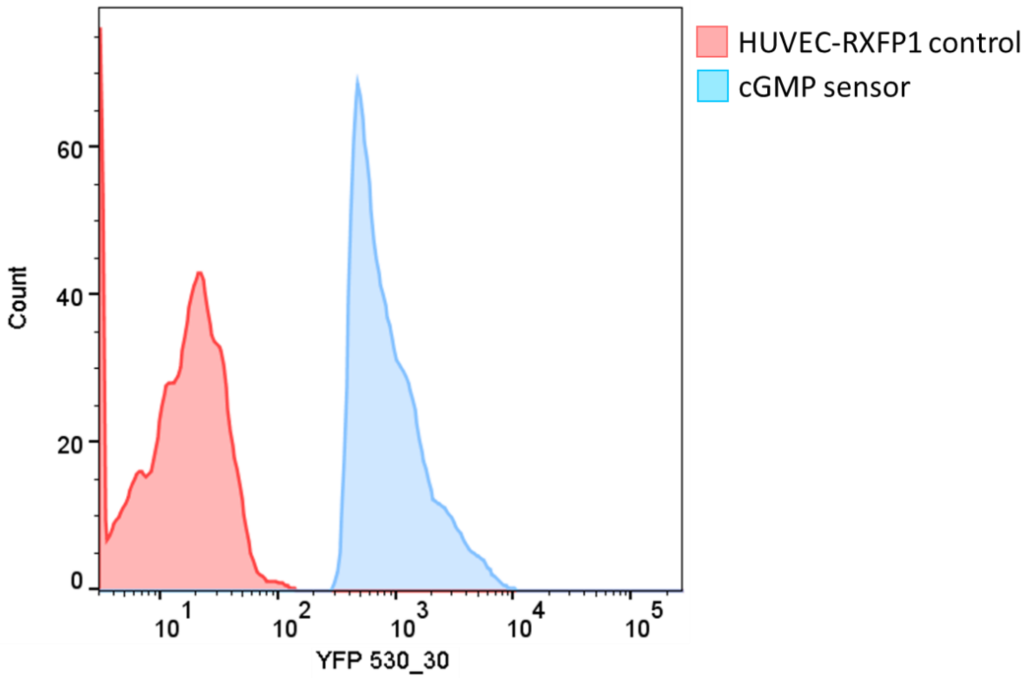

Figure S4. Representation of fluorescence-activated cell sorting (FACS) for HUVEC-RXFP1-CYGYEL cells. HUVECs expressing RXFP1 IRES GFP (HUVEC-RXFP1) were transduced with CYGYEL lentivirus and then sorted by FACS to remove non-transduced cells. Single, live cells were isolated by gating, and cells that expressed CYGYEL (based on YFP emissions that were higher than control cells) were sorted into a separate population (excitation: 488 nm; emission: 530/30 nm). As cells already expressed GFP, a 510/20 nm filter was used to detect GFP, and compensation was applied to remove GFP emissions from the YFP channel before sorting. The histogram here represents only YFP emissions of the two populations.
